# 

**Published:** 1889-01-19

**Authors:** 


**PERMANENTLY ENLARGED TO 32 PAGES.**

PRICE ONE PENNY.

No. 121, Vol. V.—January 19th, 1889.

[Registered at the General Post Office as a Newspaper.]

Per Annum, 6s. 6d., post free.

Monthly Parts, 6d.; post free, 7½d.

Ditto per Ann. 7s. 6d., post free.

### THE HOSPITAL

A WEEKLY INSTITUTIONAL JOURNAL OF

